# Childhood contact with social services and self-harm and suicidal ideation in young adulthood: A population-wide cohort study in Northern Ireland

**DOI:** 10.23889/ijpds.v8i2.2324

**Published:** 2023-09-14

**Authors:** Sarah McKenna, Dermot O'Reilly, Aideen Maguire

**Affiliations:** 1 Administrative Data Research Centre Northern Ireland, Belfast, United Kingdom; 2 Queen's University Belfast, Belfast, United Kingdom

## Objectives

Childhood contact with social services is associated with adult suicide risk, but little is known about self-harm and suicidal ideation, which are recognised predictors of suicide. This study compares self-harm (SH) and suicidal ideation (SI) in young adults with childhood history of social services contact to unexposed peers.

## Methods

A longitudinal, population-wide study of all children born 1985-1993 in Northern Ireland (NI) linking primary care registrations to social services data (1985-2015) and a national registry capturing all SH and SI presentations to the 12 Emergency Departments in NI (2012-2015). Multilevel logistic regression models estimated the association between level of contact with social services in childhood (no contact; referred but assessed as not in need (NIN); child in need (CIN); and child in care (CIC)) and SH, SI and any SH/SI, accounting for confounders and the amount of variation attributable to clustering by Health and Social Care Trust.

## Results

The cohort comprised 253,495 individuals (ages 18-30 years) alive and registered with a general practitioner during follow-up. Of the cohort, 4,026 presented with SH and 1,669 with SI. Individuals with a childhood history of social services contact comprised 10.8% of the cohort (2.9% NIN; 6.5% CIN; and 1.4% CIC) yet accounted for 40.9% of SH/SI cases. Likelihood of SH, SI, and any SH/SI increased stepwise with level of contact with social services. After full adjustment, young adults deemed NIN in childhood were three times more likely to present with SH/SI (OR 3.45 [95% CI 3.07-3.88]), former CIN five times more likely (OR 5.33 [95% CI 4.97-5.74]), and former CIC ten times more likely (OR 10.49 [95% CI 9.45-11.66]), relative to those with no contact.

## Conclusion

Adults with a childhood history of social services contact, including those assessed as not in need, account for a disproportionate number of self-harm and suicidal ideation cases. Timely and targeted interventions aimed at this population have the potential to reduce the burden of self-harm and suicide.

